# Hotspots and trends of electrochemical biosensor technology: a bibliometric analysis from 2003 to 2023†

**DOI:** 10.1039/d3ra05889a

**Published:** 2023-10-19

**Authors:** Lan Li, Yi Li, Jingwen Pei, Yu Wu, Guobing Wang, Jing Zhang, Jinbo Liu, Gang Tian

**Affiliations:** a Department of Laboratory Medicine, The Affiliated Hospital of Southwest Medical University, Sichuan Province Engineering Technology Research Center of Molecular Diagnosis of Clinical Diseases, Molecular Diagnosis of Clinical Diseases Key Laboratory of Luzhou Sichuan China tiangang@swmu.edu.cn; b Division of Basic Biomedical Sciences, The University of South Dakota Sanford School of Medicine Vermillion South Dakota 57069 USA

## Abstract

As a powerful tool for biological sensing, electrochemical biosensors have attracted much attention due to their ability to integrate biological recognition elements on an electrochemical interface and convert target analyte information into measurable electrochemical signals. Despite the abundance of literature published on the topic, no comprehensive surveys have been conducted to evaluate the area of electrochemical biosensors with bibliometric techniques. This paper employs VOSviewer to analyze and visualize literature from 2003 to 2023 in the Web of Science in order to gain an understanding of the development of the field of electrochemical biosensors in recent years. Co-occurrence and co-citation analysis are employed to identify research hotspots, trace evolutionary paths, and comprehend development trends in the field. Moreover, by analyzing highly cited and representative literature from different time periods, it is possible to recognize the major research hotspots and grasp the development pulse. The results of this study provide a comprehensive overview of the field of electrochemical biosensors and can be used to guide future research.

## Abstract 2

In this paper, the research progress of electrochemical biosensors in the last twenty years is summarized using bibliometrics, and the research hotspots and development trends are determined to guide future research.

## Graphical abstract

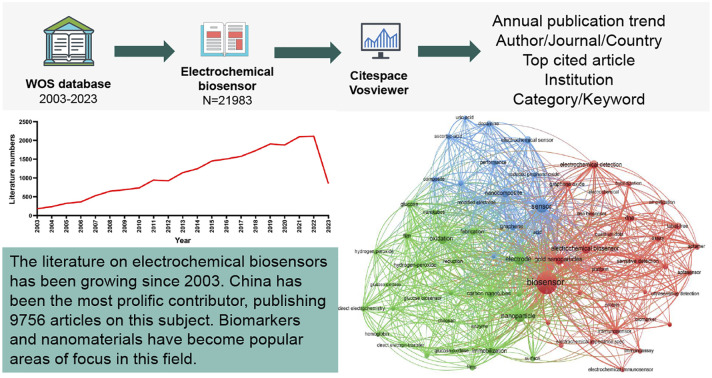

## 1. Introduction

Biosensors are composed of three main components: receptors, physicochemical transducers, and signal processors.^1,2^ Depending on the type of transducer, biosensors can be categorized into electrochemical,^3^ optical,^4^ and thermal sensitive varieties. Electrochemical sensors are particularly useful due to their low cost, easy operation, portability, fast response, and high sensitivity. Bioelectronic sensors are made by combining biological or chemical components with electronic detecting technologies and attaching physiologically sensitive elements like antigens, antibodies, enzymes, hormones, or the organism itself to electrodes. Due to the specific recognition between biomolecules, target molecules, or electroactive molecules, this enables the transformation of reaction signals into electrical signals like capacitance, current, potential, and conductivity, which in turn enables the qualitative or quantitative detection of target analytes.^5^ These features make electrochemical biosensors suitable for on-site rapid detection in clinical diagnostics,^2^ food,^6,7^ and environmental monitoring.^8^

Bibliometrics is a systematic discipline formed through the integration of mathematics, statistics, bibliography, big data analysis and other disciplines that can quantitatively analyse resources or knowledge in a certain field.^9–11^ It has important applications in various fields. It can scientifically manage publications, core documents and library information, and can also design a more time-saving and labour-saving information network system to process information efficiently.^12^ More and more medical workers use bibliometrics to carry out information management on relevant data in the medical field, capture and analyse the information of published journal documents, including the number of published papers, published authors, journal institutions, published journals, institution regions, and funding situation. Visual analysis can be used to find different types of groups by clustering the measurement indicators based on the relationship strength and direction. Further analysis of the connection and correlation degree of its highly cited documents, popular keywords, and subject terms can be done to synthesize these research results and summarize the current research status of the content.^13,14^ Moreover, bibliometric analysis has been employed in research on electrochemiluminescence and electrochemical biosensors in healthcare services.^15–17^ However, there has been no comprehensive bibliometric analysis of electrochemical biosensors reported in the literature. To bridge this gap, bibliometric analysis was employed in this study. 23 090 related literature of electrochemical biosensors were retrieved from the Web of Scientific Core Collection Database and analysed the annual publication trend, literature categories, authors, journals, articles, institutions, and keywords by VOSviewer and CiteSpace. This allowed for the summarization of the research progress of electrochemical biosensors over the past two decades, as well as the determination of the research hotspots and development trends.

## 2. Data sources and analysis methods

### 2.1. Data source

The Web of Science Core Collection database was chosen as the data source for this study due to its wide coverage of publications from different fields and its high-quality digital literature resource, which is widely regarded as the best database for bibliometric analysis.^18,19^ The search sentence used was “TS = (electrochemical biosensor)”, indexed in SSCI and ESCI, and with a time span of 2003.01.01 to 2023.06.30. No language or document type limitations were set, with the document types including original articles and review articles. After eliminating duplicated entries from the search results, a total of 24 294 journal papers were obtained. Subsequently, to make sure that the chosen articles concerned the electrochemical biosensor, a manual review was conducted to examine the content of each article (including the paper title and abstract) to exclude any irrelevant publications. Consequently, 23 090 articles were kept for content and bibliometric mapping analysis, comprising of 20 312 (88.0%) articles and 2778 (12.0%) reviews (Table 1).

**Table 1:** Summary of data source and selection

Category	Specific standard equipment
Research database	Web of science core collection
Citation indexes	SSCI and SCIE
Searching period	2003-01-01 to 2023-06-30
Search sentence	“TS = (electrochemical biosensor)”
Document types	“Articles” and “Review articles”
Data extraction	Export with full records and cited references in plain text format
Studies excluded (*N* = 1204)	Duplicates (*N* = 20) not related to electrochemical biosensor (*N* = 1184)
Sample size	23 090

### 2.2. Data analysis

GraphPad Prism (version 9.5) was utilized to generate bar charts, while VOSviewer (version 1.6.19) and CiteSpace (version win5.7. R5) were employed to conduct data visualization, creating scientific landscapes and networks based on citation frequency, countries, journals, authors, and other information.

## 3. Results

### 3.1. Annual publication trend

**Fig. 1:**
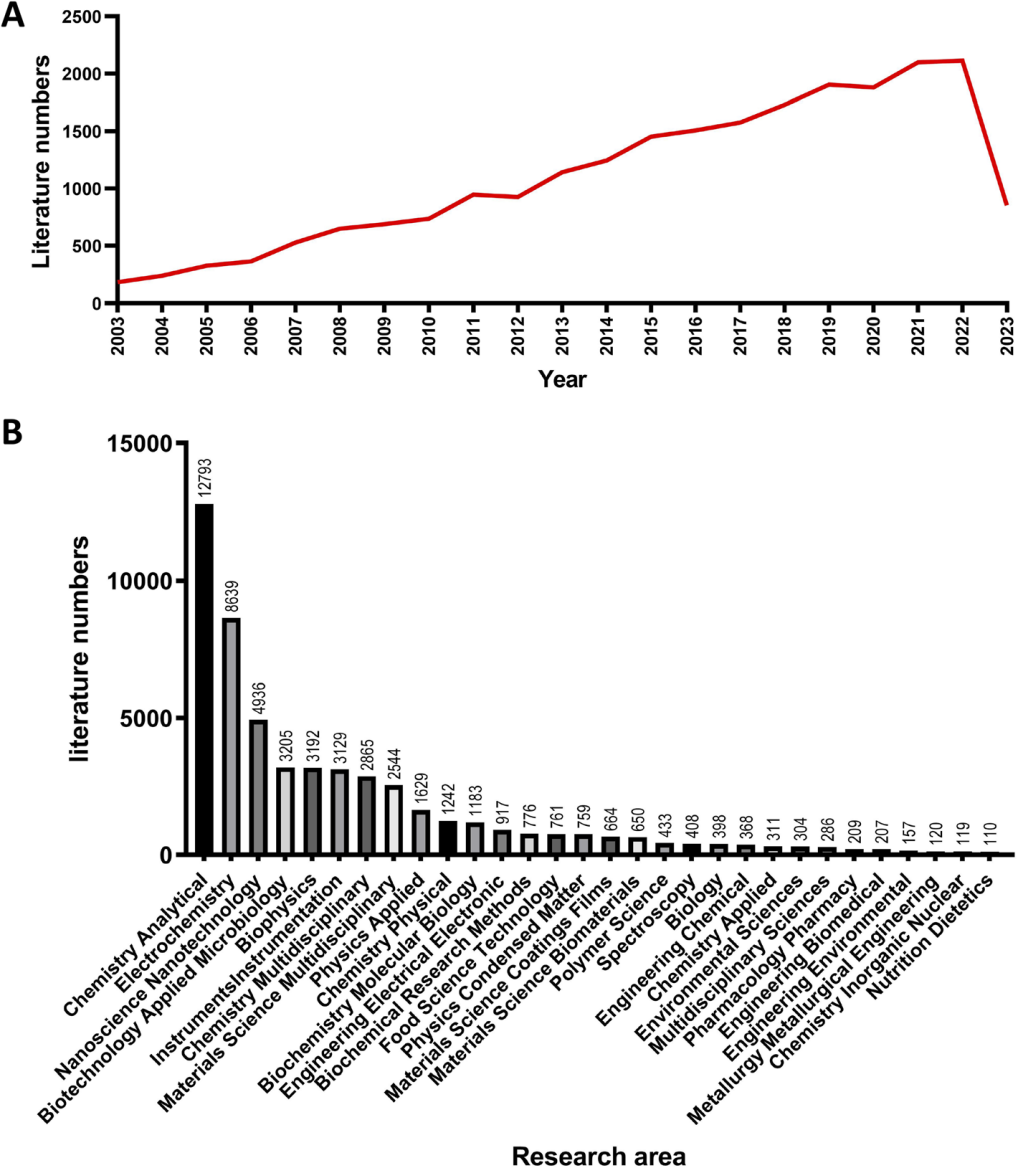
(A) Published literature on electrochemical biosensor from 2003 to 2023. (B) Published literature of electrochemical biosensor in different categories.

The data presented in Fig. 1A indicates an overall increase in the number of publications related to electrochemical biosensors from 2003 to 2023. The number of articles published is gradually increasing every year. Since 2013, the annual publication volume has been consistently above 1000 articles, reaching 2114 in 2022. As of April 30th in 2023, 854 articles have been published, and it is expected that the number of publications in 2023 will exceed 2300. This trend suggests that electrochemical biosensors have become an increasingly popular research topic, and further growth in the number of publications is likely as researchers continue to explore the molecular mechanisms and nanomaterials associated with this field.

### 3.2. Analysis of literature categories

According to Web of Science subject categories, all these literature are assigned to different research areas. Top 30 research areas ranked by publication counts are exhibited in Fig. 1B. The most involved area is the chemical analysis (54.3%) with 12 793 publications, followed by electrochemistry (8639 publications) and nanoscience (4936 publications). The number of publications related to biotechnology, applied microbiology, biophysics and instruments is roughly equivalent.

### 3.3. Analysis of authors

**Fig. 2:**
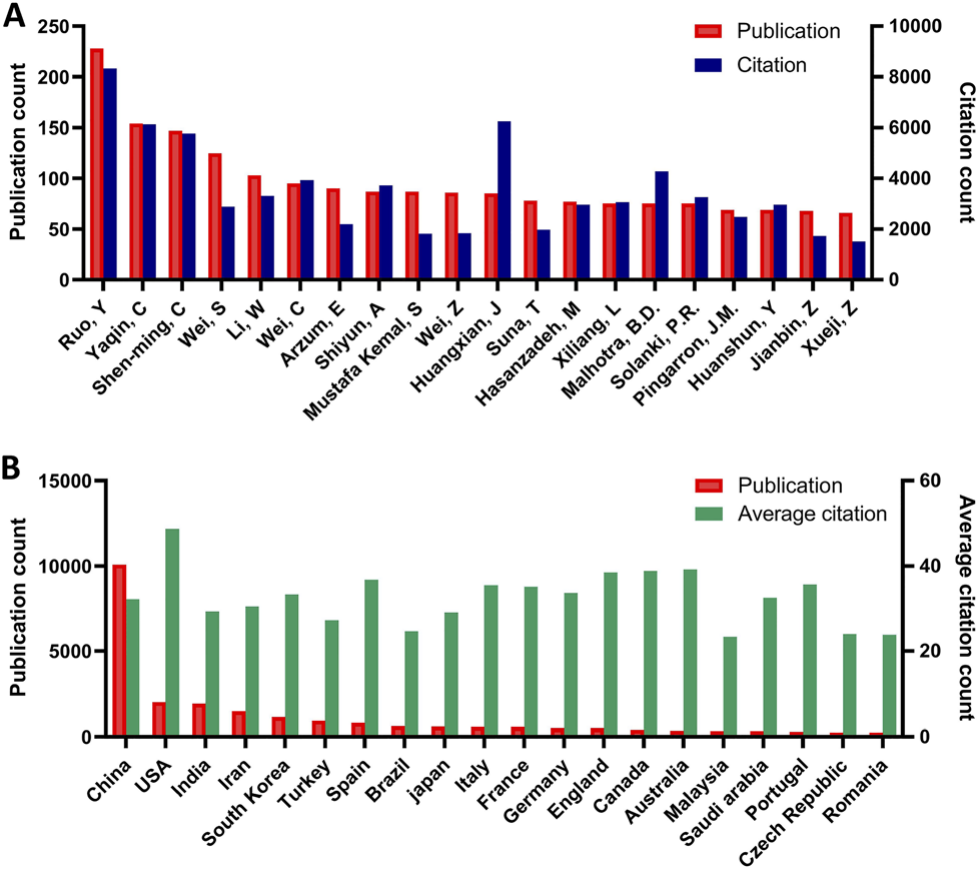
(A) Top 10 most prolific authors. (B) Top 10 most prolific countries/regions according to publication numbers.

Authors totalling 57 912 collaborated to publish 23 090 papers, with the transient authors accounting for 68.5% (39 659) of the total. 1479 authors published more than 10 articles in this field, with the most prolific author being Ruo Yuan, who published228 articles from 2003 to June 2023. Yaqin Chai was the second most prolific author, with 154 publications. The top five cited authors are Ruo, Y, Huangxian, J, Yaqin, C, Joseph, W and Shen-ming, C. The top 10 most prolific authors are presented in Fig. 2A.

**Fig. 3:**
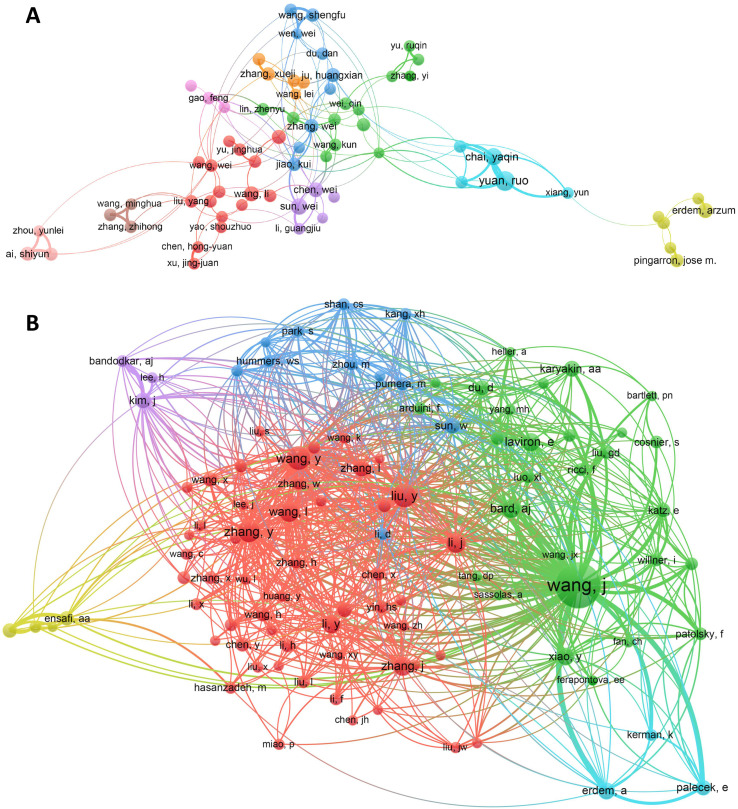
(A) Network visualization of co-authorship analysis for 68 authors with at least 40 publications. Thicker lines indicate stronger collaborations. Authors represented with larger circle size or font size had relatively more publications. (B) Network visualization of co-citation analysis for authors. Each author is represented as a node, with the size of the node being proportional to the number of citations received. Two nodes will be linked if they have been co-cited, and the proximity of the nodes will indicate the relatedness. Nodes that are closer together will be assigned to the same cluster and given the same color.

From Fig. 3A, we can see that the authors with a high volume of publications in the field of electrochemical biosensor have formed multiple cooperation networks, and the cooperation within each network is relatively close, but the cooperation between networks is relatively small. Professor Ruo Yuan and Yaqin Chai from Southwest University had a strong bond of cooperation, with a relative link strength of 151.

Analysis of author co-citation revealed that 102 authors with at least 500 citations were included. Total link strength reflects the impact of an author's documents on other authors participating in the research. As illustrated in Fig. 3B, the top three authors with the highest total link strength were Wang, J, Wang, Y, and Liu, Y.

### 3.4. Analysis of published journals

**Fig. 4:**
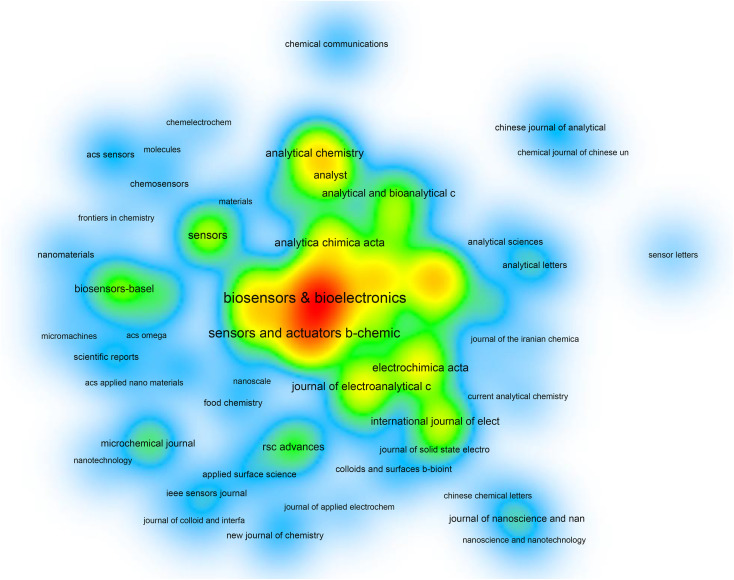
Density visualization of citation analysis for 65 journals with a minimum productivity of 50 documents. Journals with a greater amount of citations are represented by darker spots.

The research conducted has revealed that the retrieved documents were published in 1162 different journals. Table 2 highlights the 20 most active journals in publishing articles on electrochemical biosensors. *Biosensors and Bioelectronics* was the most prolific journal in this field, with 2485 publications, followed by *Sensors and Actuators B: Chemical* with 1545 publications, and *Electroanalysis* with 939 publications. The top 20 journals in total accounted for 56.6% of all documents retrieved. Citations analysis of the 369 journals with at least 5 publications indicated that *Biosensors and Bioelectronics* had the highest citation numbers (*N* = 139 852), followed by *Sensors and Actuators B: Chemical* (*N* = 58 173), and *Analytical Chemistry* (*N* = 47 103). Fig. 4 displays the density map of journals citation analysis.

**Table 2:** Top 20 prolific journals in publishing papers on electrochemical biosensor

Rank	Journal	Documents	Citations	IF (2023)
1	*Biosensors and Bioelectronics*	2485	139 852	12.600
2	*Sensors and Actuators B: Chemical*	1545	58 173	9.221
3	*Electroanalysis*	939	21 809	3.007
4	*Talanta*	775	26 979	6.556
5	*Microchimica Acta*	663	16 290	6.408
6	*Analytical Chemistry*	662	47 103	8.008
7	*Analytica Chimica Acta*	647	25 471	6.911
8	*Electrochimica Acta*	632	24 196	6.600
9	*Journal of Electroanalytical Chemistry*	614	14 835	4.598
10	*Sensors*	487	16 202	3.847
11	*International Journal of Electrochemical Science*	477	5843	1.541
12	*Journal of The Electrochemical Society*	435	5672	4.386
13	*Analyst*	432	14 107	5.227
14	*Biosensors-Basel*	381	3799	5.743
15	*Bioelectrochemistry*	361	9588	5.760
16	*Analytical Methods*	352	5288	3.532
17	*RSC Advances*	344	7258	4.036
18	*Analytical and Bioanalytical Chemistry*	311	10 655	4.478
19	*Microchemical Journal*	271	3179	5.304
20	*ACS Applied Materials & Interfaces*	245	11 320	10.383

### 3.5. Analysis of top cited articles

The 23 090 papers that were retrieved were cited 732 598 times in total. Of these, 1286 (5.6%) had at least one hundred citations, while 1852 (4.0%) had no citations. Table 3 displays the top 20 most cited papers, comprising of 12 reviews and 8 articles, coming from China (*N* = 5), USA (*N* = 5), and the remaining from South Korea, Israel, Singapore, Canada, Ireland, Spain, United Kingdom, France and Lithuania. The highest citation number of 1369 was for the article “Biological and Chemical Sensors Based on Graphene Materials” published in Chemical Society Reviews.^20^ A comprehensive review of graphene materials and graphene-based electrochemical biosensors. It is followed by the article “Wearable biosensors for healthcare monitoring” published by Kim J. *et al.* on Nature Biotechnology in 2019.^21^ An overview analysis of wearable biosensors *via* dynamic, non-invasive measurements. It can be seen from the highly cited documents that most of them are related to new materials, rapid detection and non-invasive detection, which shows that cooperation between different disciplines and technologies is necessary in order to develop forward.

**Table 3:** Top 20 cited articles on electrochemical biosensor from 2003 to 2023

Rank	Author	Title	Year	Source title	Cited by	IF	Country	Document type
1	Liu, YX *et al.*	Biological and chemical sensors based on graphene materials	2012	*Chemical Society Reviews*	1369	46.2	China	Review
2	Kim, J *et al.*	Wearable biosensors for healthcare monitoring	2019	*Nature Biotechnology*	1312	46.9	USA	Review
3	Shan, CS *et al.*	Direct electrochemistry of glucose oxidase and biosensing for glucose based on graphene	2009	*Analytical Chemistry*	1161	8.0	China	Article
4	Katz, E *et al.*	Probing biomolecular interactions at conductive and semiconductive surfaces by impedance spectroscopy: routes to impedimetric immunosensors, DNA-sensors, and enzyme biosensors	2003	*Electroanalysis*	1154	3.0	Israel	Review
5	Ronkainen, NJ *et al.*	Electrochemical biosensors	2010	*Chemical Society Reviews*	1120	46.2	USA	Review
6	Kuila, T *et al.*	Recent advances in graphene-based biosensors	2011	*Biosensors and Bioelectronics*	1013	12.6	South Korea	Review
7	Song, SP *et al.*	Aptamer-based biosensors	2008	*TrAC Trends in Analytical Chemistry*	1003	13.1	China	Article
8	Pumera, M *et al.*	Graphene for electrochemical sensing and biosensing	2010	*TrAC Trends in Analytical Chemistry*	964	13.1	Singapore	Review
9	Hrapovic, S	Electrochemical biosensing platforms using platinum nanoparticles and carbon nanotubes	2004	*Analytical Chemistry*	925	8.0	Canada	Article
10	Daniels, JS *et al.*	Label-free impedance biosensors: opportunities and challenges	2007	*Electroanalysis*	893	3.0	USA	Review
11	Lin, YH *et al.*	Glucose biosensors based on carbon nanotube nanoelectrode ensembles	2004	*Nano Letters*	834	10.8	USA	Article
12	Jacobs, CB *et al.*	Review: carbon nanotube based electrochemical sensors for biomolecules	2010	*Analytica Chimica Acta*	805	6.9	USA	Review
13	Chen, W *et al.*	Recent advances in electrochemical sensing for hydrogen peroxide: a Review	2012	*Analyst*	787	5.2	China	Review
14	Velusamy, V *et al.*	An overview of foodborne pathogen detection: In the perspective of biosensors	2010	*Biotechnology Advances*	780	16.0	Ireland	Review
15	Pingarron, JM *et al.*	Gold nanoparticle-based electrochemical biosensors	2008	*Electrochimica Acta*	756	6.6	Spain	Review
16	Chang, BY *et al.*	Electrochemical impedance spectroscopy	2010	*Annual Review of Analytical Chemistry*	723	12.4	South Korea	Article
17	Toghill, KE *et al.*	Electrochemical non-enzymatic glucose sensors: a perspective and an evaluation	2010	*International Journal of Electrochemical Science*	716	1.5	United Kingdom	Article
18	Sassolas, a *et al.*	Immobilization strategies to develop enzymatic biosensors	2012	*Biotechnology Advances*	708	16.0	France	Review
19	Ramanavicius, a *et al.*	Electrochemical sensors based on conducting polymer-polypyrrole	2006	*Electrochimica Acta*	696	6.6	Lithuania	Article
20	Guo, SJ *et al.*	Platinum nanoparticle ensemble-on-graphene hybrid nanosheet: one-pot, rapid synthesis, and used as new electrode material for electrochemical sensing	2010	*ACS Nano*	693	17.1	China	Article

### 3.6. Analysis of countries/regions

**Fig. 5:**
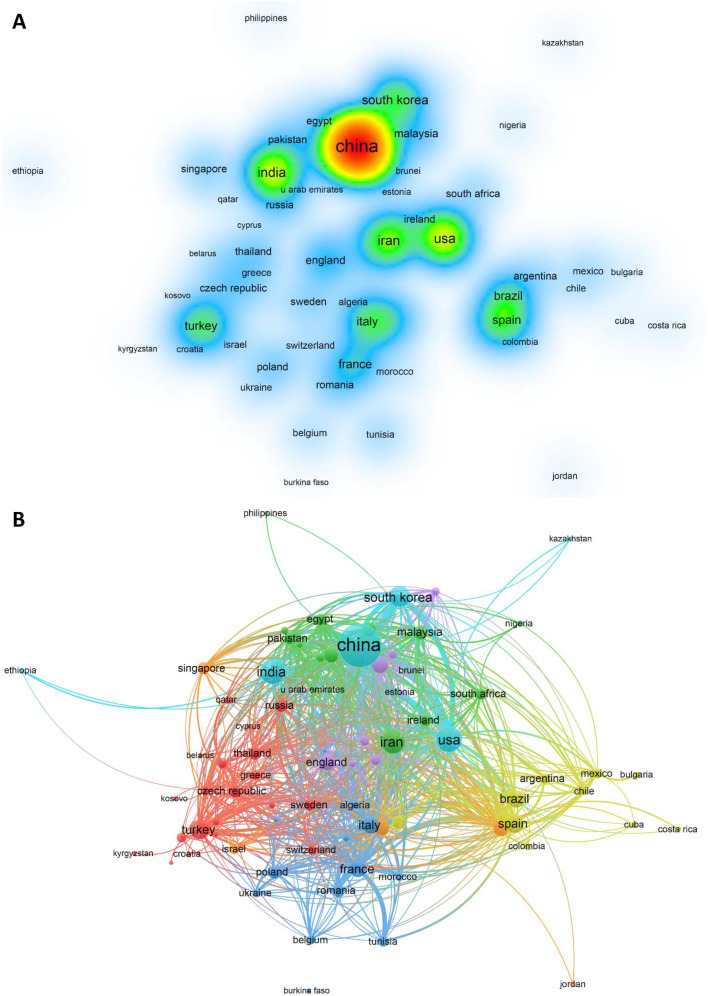
(A) Density visualization of citation analysis for countries with a minimum citation of 100, a total of 78 countries and regions meet the threshold. Nations with the highest citation counts are represented by darker spots. (B) Network visualization of co-authorship analysis for countries. Nodes are used to represent each country, with the size of the node being proportional to the sum of its publications.

120 countries/regions were represented in the geographical distribution of publications. 15 countries only published one article, while 71 countries published more than ten. It is evident from Fig. 2B that China is the most productive country in terms of the number of published articles, accounting for 43.7% of the total, significantly higher than the second-ranked USA with 2002 articles. Among the top 20 most prolific countries, United States had the greatest citation numbers per publication (48.7), followed by Australia (39.2) and Canada (38.9). Fig. 5A further illustrates that China has the most citations, far exceeding other countries, followed by the United States, India, Iran, and South Korea. Fig. 5B provides a visualization map of the country co-authorship analysis conducted by VOSviewer, which indicates that China is situated in the centre of the network. The presented node connecting line portrays the intensity of the association, where a thicker line indicates the greater number of articles released through collaboration of two countries. As per the collaboration analysis, China is seen to have the highest degree of centrality, followed by the USA, with both countries exhibiting a close cooperation. Similarly, India, Iran and South Korea have also developed a strong connection. Close cooperation and exchanges between countries contribute to the rapid development of this field.

### 3.7. Analysis of institutions

**Fig. 6:**
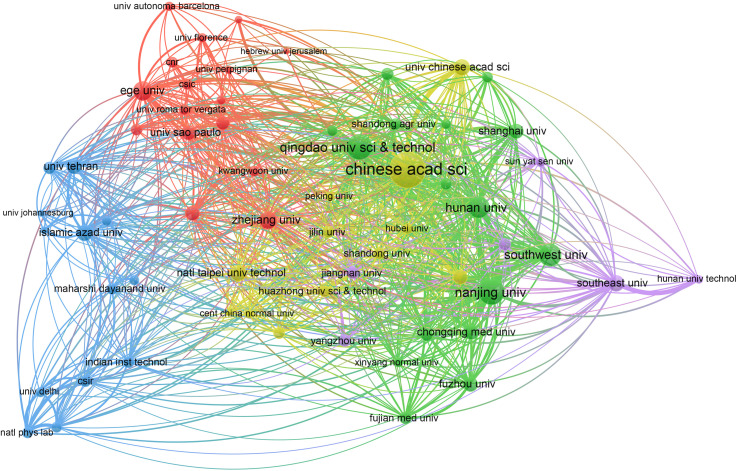
Network visualization of citation analysis for institutions. Each institution is represented as a node, with the size of the node proportional to the sum of citations. Links between two nodes indicate a citation relationship, and the distance between them reflects the relatedness; nodes that are closer together are more likely to be grouped together with the same color.

As shown in Table 4, the Chinese Academy of Sciences in China was the most prolific institution in the publication of papers on electrochemical biosensors, with 774 publications. This was followed by Qingdao University of Science & Technology in China (449 publications) and Nanjing University in China (434 publications). Of the top 10 prolific institutions, 8 were in China, 1 was in Turkey, and 1 was in Iran. Additionally, 63 organizations were cited at least 3000 times, as shown in Fig. 6. The Chinese Academy of Sciences had the highest citation number with 36 245 citations, followed by Nanjing University (25 551 citations) and Qingdao University of Science & Technology (13 621 citations).

**Table 4:** Top 10 prolific institutions in publishing papers on electrochemical biosensor

Rank	Organization	Documents	Country
1	Chinese Academy of Sciences	774	China
2	Qingdao University of Science & Technology	449	China
3	Nanjing University	434	China
4	Southwest University	344	China
5	Hunan University	317	China
6	Ege University	262	Turkey
7	Zhejiang University	224	China
8	Southeast University	199	China
9	Islamic Azad University	194	Iran
10	University of Chinese Academy of Sciences	193	China

### 3.8. Analysis of keywords

**Fig. 7:**
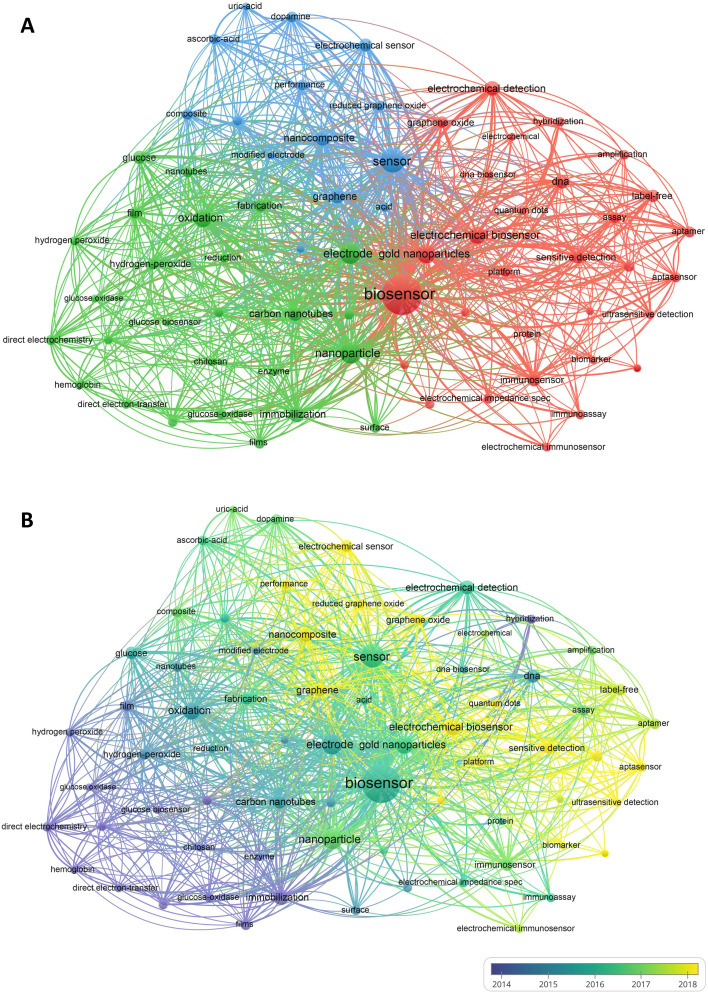
(A) Network visualization of co-occurrence analysis for keywords. Clustering of the top 70 keywords with the highest number of occurrences. The size of the nodes symbolizing the amount of articles in the electrochemical biosensor field that contain the keywords. Furthermore, the connecting lines between the nodes depict the relationship between the keywords. (B) Overlay visualization of co-occurrence analysis for keywords.

The analysis of keyword co-occurrence revealed the research hotspots in this field.^15^Table 5 lists the top 10 keywords with the highest frequency of occurrence, with “biosensor” being the most mentioned. Fig. 7A illustrates the three clusters identified among the top 70 keywords, based on the frequency of co-occurrence. The cluster led by “biosensor” and “gold nanoparticles” had the highest number of occurrences, followed by the cluster led by “sensor” and “nanoparticles”, and then the cluster led by “oxidation” and “immobilization”. Fig. 7B is the keyword overlay map, showing the trend of keywords over time. The yellow nodes depicted in the figure signify emerging keywords, suggesting that they could be indicative of ongoing research studies; such keywords include sensitive detection, ultrasensitive detection, rapid detection, nanocomposite, and signal amplification, all of which have been frequently cited in the last two years and may be potential research hotspots in the future. The network visualization of keywords of the top five most prolific countries are shown in Fig. S1–S5.† The keywords in the five countries are basically the same, and the top five are “biosensor”, “sensor”, “nanoparticle”, “electrode” and “gold nanoparticles”.

**Table 5:** Top 10 Keywords of electrochemical biosensor between 2003 and 2023

Rank	Keyword	Occurrences	Cluster
1	Biosensor	11 917	1
2	Sensor	4816	3
3	Nanoparticle	3869	3
4	Electrode	3513	3
5	Gold nanoparticles	2825	1
6	Oxidation	2656	2
7	Electrochemical biosensor	2470	1
8	Immobilization	2069	2
9	Graphene	1728	3
10	Carbon nanotubes	1674	2

**Fig. 8:**
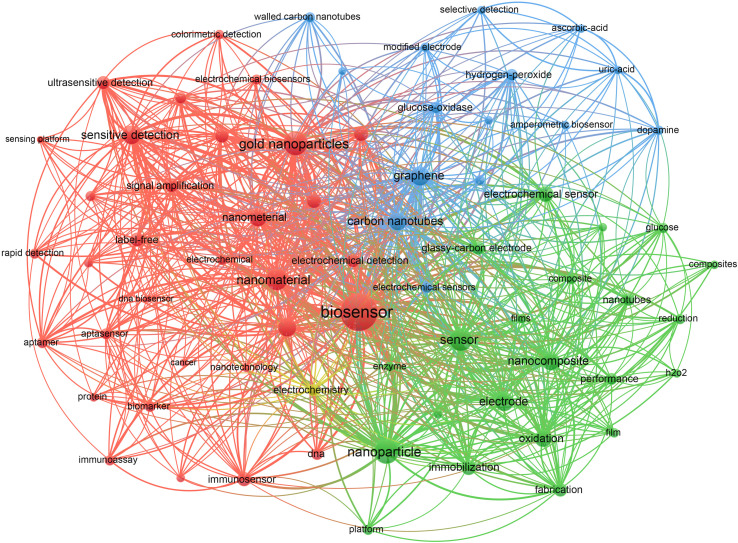
Network visualization of co-occurrence analysis for keywords of nanomaterials. Clustering of the top 70 keywords with the highest number of occurrences. The size of the nodes symbolizing the amount of articles in the electrochemical biosensor field that contain the keywords. Furthermore, the connecting lines between the nodes depict the relationship between the keywords.

To further analyse the application of nanomaterials in electrochemical biosensors, we conducted keyword analysis on 717 articles using the search formula “(TS = (electrochemical biosensor)) ANDTS = (nanomaterial)”, as shown in Fig. 8. The results indicate that nanomaterials such as gold nanoparticles, graphene, and carbon nanotubes are the most commonly utilized in the field of electrochemical biosensors.

**Fig. 9:**
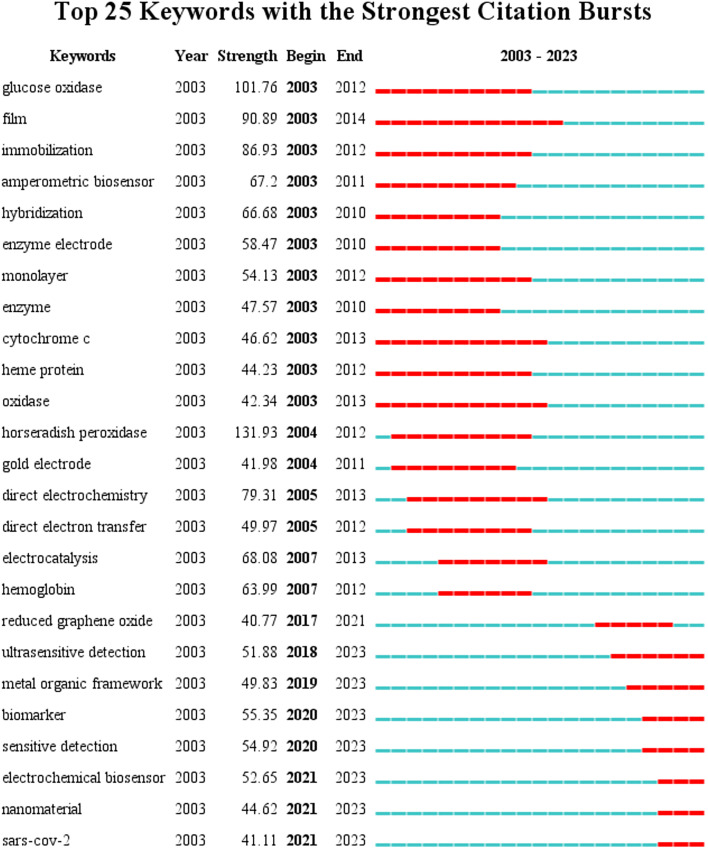
Top 25 keywords with the strongest citation bursts.

Keyword burst detection is a useful tool for identifying research frontiers and emerging topics in a particular field over time.^22^ A burst keyword is one that appears frequently in a given period of time, providing insight into the evolution of research hotspots and trends. To gain a better understanding of the development of electrochemical biosensor research, the top 25 keyword terms with the strongest emergent strength analysed from 2003 to 2023 are illustrated in Fig. 9. Biomarkers and their associated characteristics have undoubtedly become a subject of great interest among scholars since 2020, and will likely remain a point of focus in the future. Biomarkers are biological substances or activities that can be quantitatively measured and evaluated to indicate normal biological processes, pathogenic processes, or responses to therapeutic interventions. Recently, electrochemical biosensors have been increasingly employed for the detection of biomarkers. Since 2019, the spread of Coronavirus Disease (COVID-19), caused by Severe Acute Respiratory Syndrome Coronavirus 2 (SARS-CoV-2), has been a global concern, resulting in numerous fatalities and significant economic losses.^23,24^ As a result, the development of specific and sensitive detection methods for COVID-19 has become a priority.^25^ Additionally, nanomaterials are being explored as a potential future research trend, as nanobiosensors can provide high sensitivity and portability, potentially leading to the development of new clinical and field-deployable analytical instruments.^26,27^

## 4. Discussion

Electrochemical biosensors, as a growing and useful detection method, have been continually improved with the advancement of the medical field. This study utilizes VOSviewer software to review the development of electrochemical sensors in the past two decades, analysing the core authors, high-productivity international institutions, key journals, and keywords in the field. Our findings indicate that the amount of literature on electrochemical biosensors is on an overall upward trend, from 183 articles in 2003 to 2114 articles in 2022, with the number of articles increasing 11.5 times. Similar to our findings, prior bibliometric analyses have demonstrated that the amount of literature on biosensors and electrochemiluminescence sensing technology is growing in general.^15,17^ It is clear that electrochemical biosensors are a gradually growing field.

It is noteworthy that Joseph Wang from the University of California had a central position in the co-citation map, despite having a relatively small number of publications. This could be attributed to several highly-cited publications from him.^21,28^ Wang's research interests focus on electrochemical biosensors, wearable devices and microrobots. Similarly, Huangxian, J published only half as many articles as Yaqin, C, but her citations were higher than Yaqin, C. This result suggests that the number of publications may not always be an accurate indicator of an author's academic influence, as there are many factors that can influence an article's citation frequency. Publications of Chinese scholars occupied the leading position, but its average citation rate ranks 12th. This is because quantity remains the measuring stick at the university and the national levels.^29^ Reward and promotion systems which eschew quantity measures and value fewer, deeper, more novel contributions could inspire more innovative and high-quantity work.^30^

Research on national cooperation networks can help to promote teamwork and global cooperation in specific areas. In this study, we can see that the country with the highest number of publications is China, followed by the USA and India. Furthermore, China has the highest degree of centrality, indicating that it plays an important role as a bridge in intercountry cooperation. Cooperation as a conventional development modality, which sharing resources has become a trend in global research. Based on this current situation, strengthening communication and cooperation between national institutions is of profound importance in promoting further development in this field. The analysis of author collaboration networks and author co-citations helps to analyse the direction of authors' research and provides further guidance. The study shows that many scholars have formed collaborative networks centred on themselves and have a more consistent article output. Overall, there is a lack of collaboration between scholars and teams, and enhanced communication and cooperation between authors could have far-reaching implications in promoting further development in the field. Future author collaboration could produce more good quality articles.

Electrical signal changes are the basis of signal production in electrochemical biosensing systems, which can be detected by changes in current and resistance. The majority of electrochemical biosensor studies are classified as chemistry analytical and electrochemistry due to the presence of oxidation–reduction processes.

Electrochemical biosensors have been widely utilized for clinical diagnosis, food analysis, and environmental monitoring, and they have made remarkable progress in detecting not only bacteria, hormones, and total metal ions, but also disease biomarkers, which has become a popular research topic in recent years and is believed to be an effective approach for exploring disease biology and managing diseases.^31^ Biomarkers are molecular indicators of a biological state or condition, which can be proteins, protein fragments, DNA/RNA, or organic chemicals produced by abnormal cells. Disease biomarkers are essential for the early detection and accurate staging of a disease, as they serve as a molecular representation of the physiological state of the disease at a given time.^32^ Disease biomarkers, due to their ability to provide information on the initiation of a disease, allow for powerful methods for diagnosis and treatment. Electrochemical biosensors are advantageous due to their convenience and timely analysis, making them key components in biomarker detection. In the future, the combination of biomarkers discovered through mechanistic studies, biochemical analysis, and other methods with rapid and accurate electrochemical biosensors will be an essential research trend.

As medicine continues to advance, the demand for trace biomolecule detection and point-of-care diagnostics has increased, making the simplification of operation procedures and shortening of detection time for analytical methods increasingly important. The fabrication of electrochemical biosensors with superior selectivity, high sensitivity, and fast response is of great importance. Signal amplification is a commonly used method to raise the detection sensitivity in biosensors. With the advancement of functional materials, nanomaterials and nanotechnology have been extensively studied by scientists in various domains. The advancement of nanotechnology has been remarkable, and nanomaterials have been highly sought after for their exceptional properties, leading to the development of nanomaterial sensors with remarkable sensitivity and accuracy for diagnostic purposes, thus improving the performance of biosensors in terms of sensitivity, detection limit, selectivity and repeatability.^33,34^ According to the analysis of the application of nanomaterials in electrochemical biosensors, graphene, carbon nanotubes and gold nanoparticles have displayed exceptional capabilities for electrochemical biosensing. Among the top 20 highly cited articles, nine articles are all related to nanomaterials.

From a scientific bibliometrics perspective, the research lineage produced from this study is immensely beneficial, offering researchers insight into potential research paths and ideas in this area. Specifically: (1) it can help scholars who are interested in the application of electrochemical biosensors to establish a clear framework of the existing research in this field, and gain an in-depth understanding of the development process of this field; (2) the clustering of high-frequency keywords and the analysis of the evolution of the keywords can help scholars to understand the focus and hotspot of the field of electrochemical biosensors, which can provide scholars with references for choosing the topics of research; (3) the analysis of core papers and core authors in the field of electrochemical biosensors can help scholars quickly find the literature they want to refer to in their research; (4) the analysis of journals in this field can provide scholars with a guide to select journals to a certain extent when they submit their papers in this topic.

This paper has certain limitations, as the papers selected for the international study were only screened from the two major indexes of the Web of Science database, and other scientific databases (*e.g.*, Scopus) were not taken into account. This may lead to an incomplete analysis of the data, thus the next study should expand the scope of the literature screening to gain a more comprehensive understanding of the research and the cutting-edge hotspots in the field of electrochemical biosensors. This will help to improve and deepen the knowledge of the field, while also avoiding as much as possible the subjectivity of personal analysis and interpretation, in order to provide a more reliable review and analysis. In addition, since the number of citations of a document is affected by its publication time, the citations of high-quality literature published in 2023 may not have reached the ideal level, which may lead to a delay in the discovery of research fronts.

## 5. Conclusions

In the current age, biosensors have been extensively used in a variety of fields, including biological diagnostics, environmental monitoring, food safety, drug research, and tracking the progression of diseases at the point-of-care. In the last two decades, China has been the leading contributor to electrochemical biosensor research, with more than half of the top 10 most productive institutions and authors being from the country. Most of the research papers were published in methodological journals, with the main focuses being biomarker, ultrasensitive detection, and nanomaterial.

## Author contributions

Lan Li: conceptualization, methodology, software, writing – original draft. Yi Li: data curation, writing – original draft preparation. Jingwen Pei: visualization, data curation. Yu Wu: formal analysis, software. Guobing Wang: software, validation. Jing Zhang: writing – reviewing and editing. Jinbo Liu: writing – reviewing and editing, supervision. Gang Tian: supervision, funding acquisition. All authors have read and approved the manuscript.

## Conflicts of interest

The authors declare that the research was conducted in the absence of any commercial or financial relationships that could be construed as a potential conflict of interest.

## Supplementary Material

RA-013-D3RA05889A-s001

RA-013-D3RA05889A-s002
