# Supplementary material for: Hotspots and trends of electrochemical biosensor technology: a bibliometric analysis from 2003 to 2023
Source: RSC Adv. 2023 Oct 19;13(44):30704–17. doi: 10.1039/d3ra05889a (PMC10585615; doi:10.1039/d3ra05889a)
Supplement: RA-013-D3RA05889A-s002 [file RA-013-D3RA05889A-s002.pdf]

Electronic Supplementary Material (ESI) for RSC Advances.
This journal is © The Royal Society of Chemistry 2023

Supplementary figures

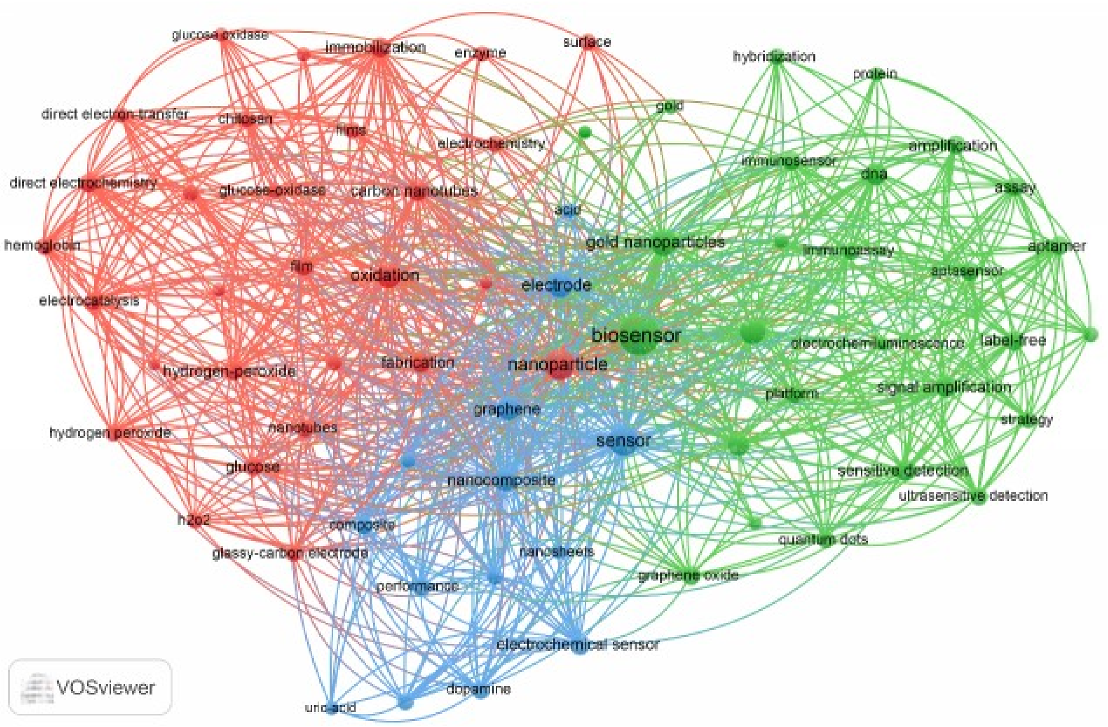

Fig. S1. Network visualization of co-occurrence analysis for keywords of 10086 Chinese articles. Clustering of the top 70 keywords with the highest number of occurrences.

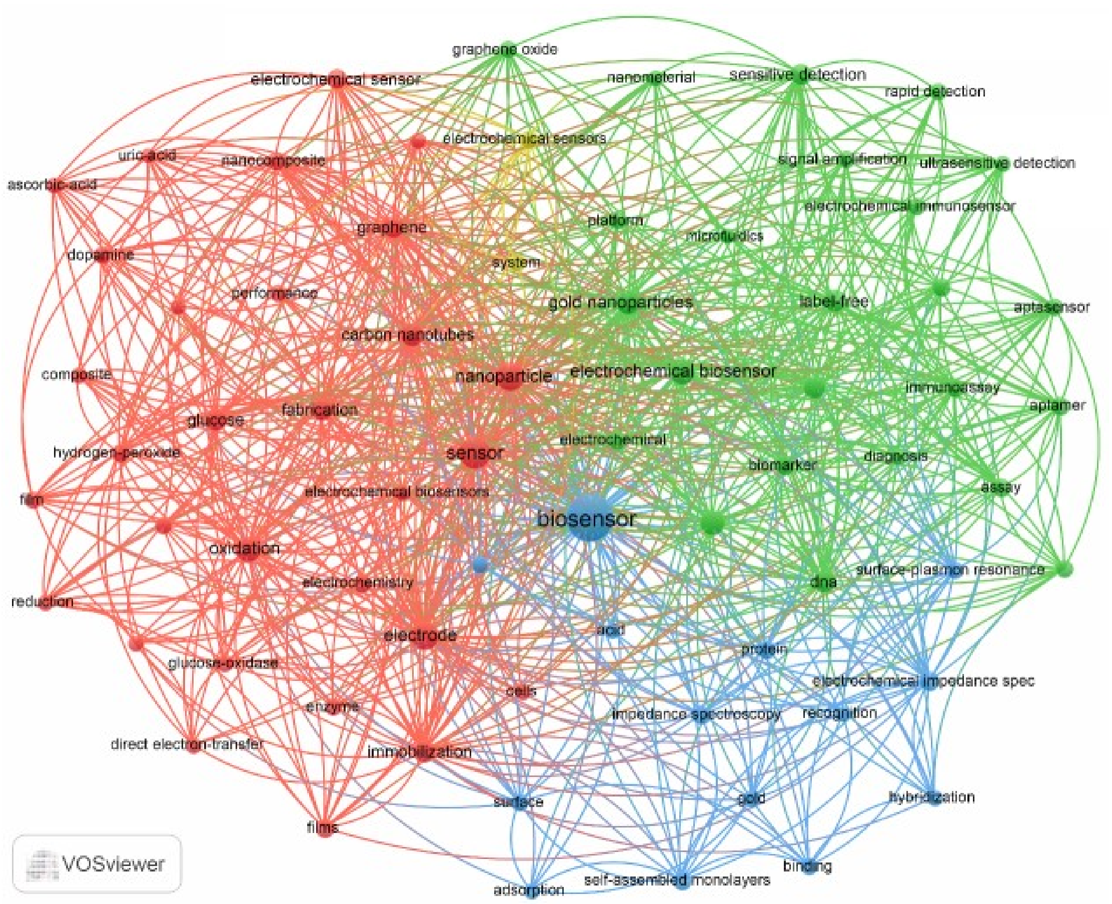

Fig. S2. Network visualization of co-occurrence analysis for keywords of 2002 American articles. Clustering of the top 70 keywords with the highest number of occurrences.

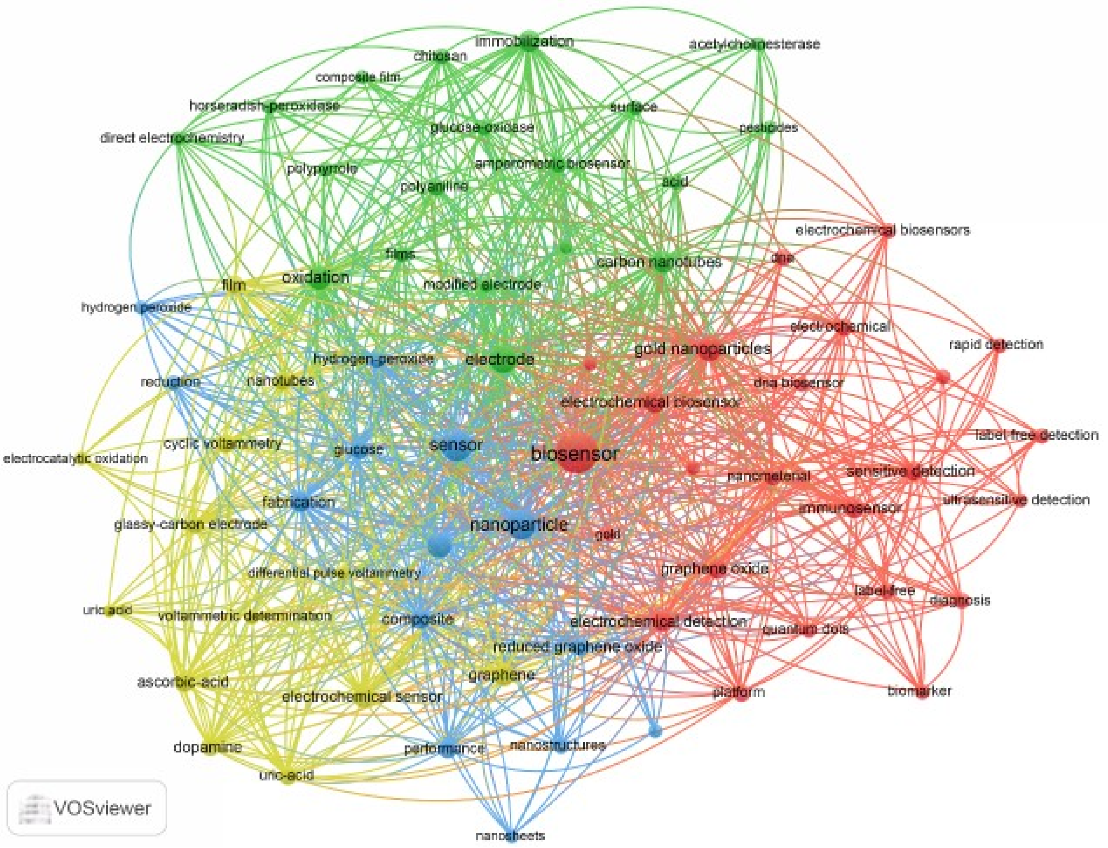

Fig. S3. Network visualization of co-occurrence analysis for keywords of 1918 Indian articles. Clustering of the top 70 keywords with the highest number of occurrences.

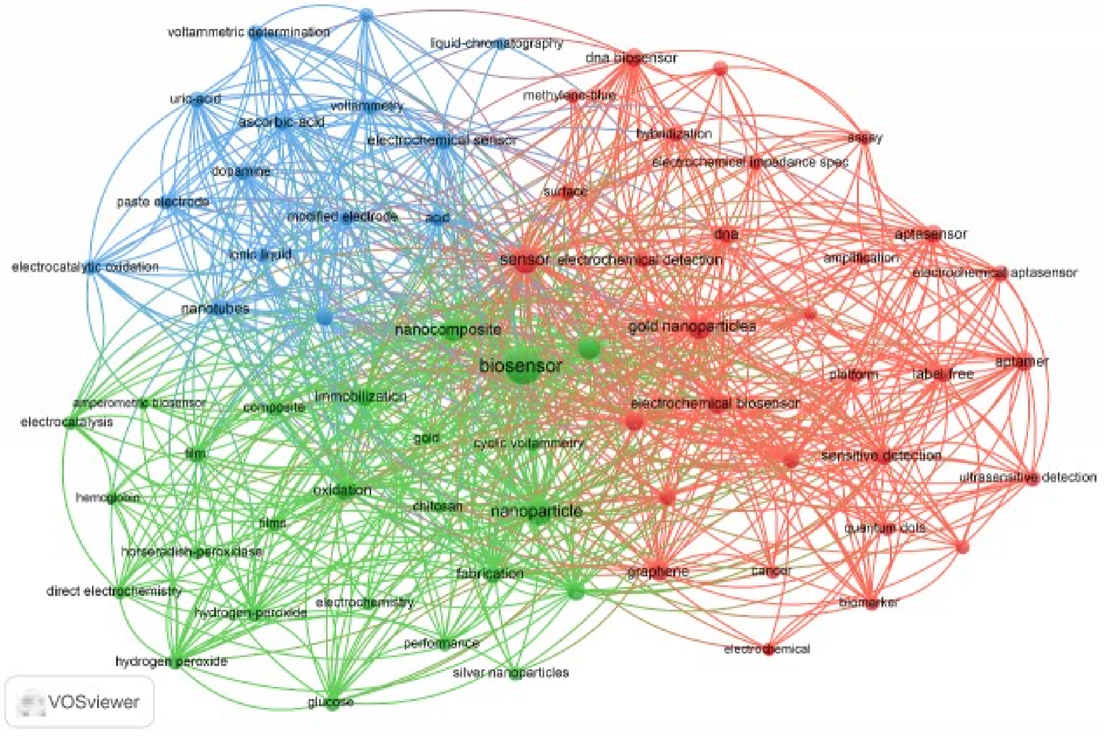

Fig. S4. Network visualization of co-occurrence analysis for keywords of 1486 Iranian articles. Clustering of the top 70 keywords with the highest number of occurrences.

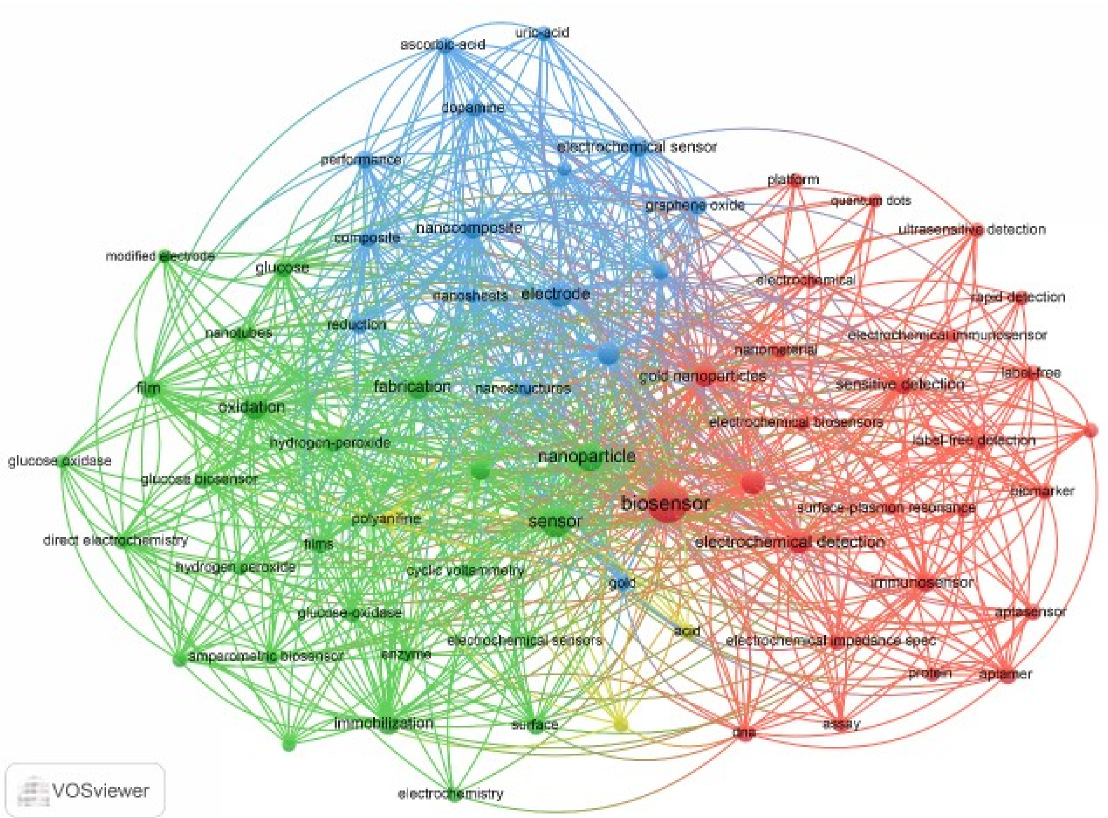

Fig. S5. Network visualization of co-occurrence analysis for keywords of 1144 Korean articles. Clustering of the top 70 keywords with the highest number of occurrences.
